# Regulation of the Immune Balance During Allogeneic Hematopoietic Stem Cell Transplantation by Vitamin D

**DOI:** 10.3389/fimmu.2019.02586

**Published:** 2019-11-05

**Authors:** Cindy Flamann, Katrin Peter, Marina Kreutz, Heiko Bruns

**Affiliations:** ^1^Department of Internal Medicine 5, Hematology/Oncology, Friedrich Alexander University Erlangen–Nuremberg, Erlangen, Germany; ^2^Department of Internal Medicine III – Hematology and Internal Oncology, University Hospital of Regensburg, Regensburg, Germany

**Keywords:** vitamin D, GvH, GvL, immune balance, macrophages, T cells, infection

## Abstract

One of the most promising therapeutic approaches for numerous hematological malignancies represents the allogeneic hematopoietic stem cell transplantation (allo-HSCT). One major complication is the development of the life-threatening graft-vs.-host disease (GvHD) which limits beneficial effects of graft-vs.-leukemia (GvL) responses during allo-HSCT. Strengthening GvL effects without induction of severe GvHD is essential to decrease the relapse rate after allo-HSCT. An interesting player in this context is vitamin D_3_ since it has modulatory capacity in both preventing GvHD and boosting GvL responses. Current studies claim that vitamin D_3_ induces an immunosuppressive environment by dendritic cell (DC)-dependent generation of regulatory T cells (Tregs). Since vitamin D_3_ is known to support the antimicrobial defense by re-establishing the physical barrier as well as releasing defensins and antimicrobial peptides, it might also improve graft-vs.-infection (GvI) effects in patients. Beyond that, alloreactive T cells might be attenuated by vitamin D_3_-mediated inhibition of proliferation and activation. Despite the inhibitory effects of vitamin D_3_ on T cells, anti-tumor responses of GvL might be reinforced by vitamin D_3_-triggered phagocytic activity and antibody-based immunotherapy. Therefore, vitamin D_3_ treatment does not only lead to a shift from a pro-inflammatory toward a tolerogenic state but also promotes tumoricidal activity of immune cells. In this review we focus on vitamin D_3_ and its immunomodulatory effects by enhancing anti-tumor activity while alleviating harmful allogeneic responses in order to restore the immune balance.

## Introduction

The most promising curative therapeutic strategy for a broad spectrum of hematological malignancies remains the allogeneic hematopoietic stem cell transplantation (allo-HSCT) (1). Its efficacy is mainly mediated by alloreactive donor-derived immune cells eliminating malignant host cells, a process known as graft-vs.-leukemia (GvL) effect (2). However, infused donor cells can also attack healthy host tissues due to histocompatibility mismatches, which leads to graft-vs.-host disease (GvHD). This life-threatening complication limits the beneficial effects mediated by GvL. Restoring the host's immune balance during and after transplantation is one of the major challenging obstacles in clinical research (3). Alleviating GvHD responses while boosting anti-leukemia activities could be the key to successful treatment in allo-HSCT. Since both processes underlie more or less the same T cell activity, it is very demanding to dissect GvHD from GvL effects (4). The current standard treatment consists mainly of corticosteroids and calcineurin inhibitors such as cyclosporine and tacrolimus (5). Since these immunosuppressive drugs attenuate T-cell-mediated inflammation (6) and the allo-stimulatory capacity of DCs (7), they lead to alleviation of GvHD symptoms. However, these immunosuppressive mechanisms might reduce GvL effects as well. Recent studies have established promising strategies for strengthening GvL responses without exacerbating GvHD. Infusion of donor lymphocytes, CAR-T cells and checkpoint inhibitors have gained pivotal interest in clinical studies over the past years (8). However, none of these therapeutic approaches target both GvHD and GvL.

**Figure 1:**
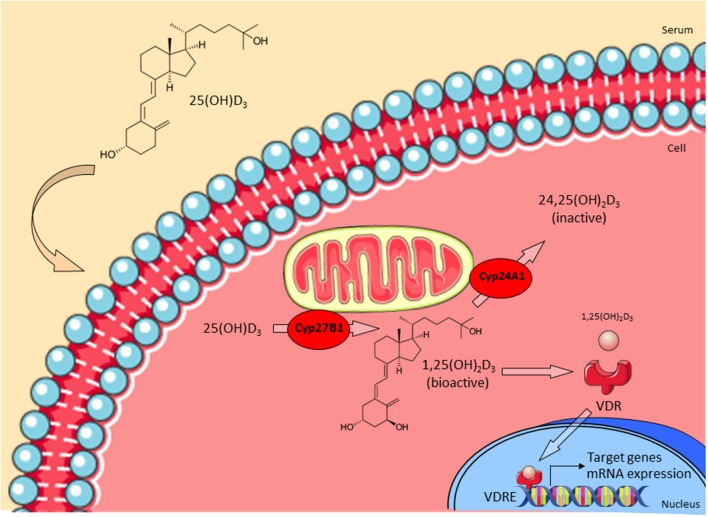
Vitamin D_3_ metabolism. The precursor form of vitamin D_3_ 25(OH)D_3_ enters the cell and is then converted into the bioactive form 1,25(OH)_2_D_3_ by CYP27B1. CYP24A1 regulates levels of 1,25(OH)_2_D_3_ by converting it into the inactive 24,25(OH)_2_D_3_. Active 1,25(OH)_2_D_3_ binds to the vitamin D_3_ receptor (VDR) in the cytoplasm and this complex translocates into the nucleus. Finally, VDR binds to appropriate vitamin D response elements (VDRE) and triggers transcription of target genes (e.g., LL-37). Adapted from Bruns and Stenger (14).

Though vitamin D_3_ has been discovered as an important regulator of calcium homeostasis in the early Twentieth century, its putative immunoregulatory role remained undiscovered until recently (9). Contrary to initial assumptions that vitamin D_3_ is mainly produced in kidney and liver, vitamin D_3_ receptor (VDR) and vitamin D_3_ metabolizing enzymes are also expressed in various types of immune cells (10–12). The novel role of vitamin D_3_ in regulating effector functions of human macrophages is closely linked to the expression of the vitamin D-1-hydroxylase CYP27B1. The precursor form of vitamin D_3_ is produced in the epidermis upon ultraviolet B (UVB) irradiation or obtained from dietary intake (13). Vitamin D_3_-binding protein (VDBP) binds pre-vitamin D_3_ and is responsible for its transport into the liver. Upon entering the cell, CYP27B1 catalyzes the conversion of 25-hydroxy-vitamin D_3_ (25(OH)D_3_) into its bioactive form 1,25-dihydroxy-vitamin D_3_ (1,25(OH)_2_D_3_, calcitriol) (14). Levels of 1,25(OH)_2_D_3_ are regulated by the inactivating 1,25(OH)_2_D_3_ 24-hydroxylase (CYP24A1). 1,25(OH)_2_D_3_ binds intracellularly to VDR and induces as a transcription factor the expression of a broad variety of target genes which contain vitamin D_3_ response elements (VDRE) within their promoters (15) (Figure 1).

Since vitamin D_3_ is well-known for exerting both anti-tumoricidal and anti-inflammatory functions, it might be an attractive target for preservation of the immune balance in patients undergoing allo-HSCT (16). In this review, we seek to elucidate mechanisms by which vitamin D_3_ might act as potential immune regulator in GvL as well as GvHD while highlighting its effects on both innate and adaptive immune system.

## Vitamin D_3_ and GvHD

At the beginning of the Twenty-first century, vitamin D_3_ has gained more attention in the field of allo-HSCT. Given that vitamin D_3_ exerts “non-classical” actions besides sustaining bone metabolism and calcium homeostasis, paved the way for pioneering studies which proved that vitamin D_3_ deficiency correlates directly with immune diseases such as multiple sclerosis (MS) (17), systemic lupus erythematosus (18), inflammatory bowel disease (IBD) (19), rheumatoid arthritis (20), and autoimmune thyroid disease (21). Vitamin D_3_ supplementation has been shown to reduce severity and incidence of such diseases not only in animal models but also in clinical studies (22). Based on studies which show that application of several vitamin D_3_ analogs has been effective in some solid organ transplantations (23–25), Pakkala and colleagues successfully achieved prevention of GvHD symptoms in a rat transplantation model by a 1,25(OH)_2_D_3_ analog (MC1288) (26). Further investigations proved that certain VDR polymorphisms are associated with higher risk of severe GvHD (27–29). Since patients receiving HSCT are malnourished, less exposed to sunlight and have an altered vitamin D_3_ metabolism due to medications and impaired organ function, they are predestined for vitamin D_3_ deficiency (30). In fact, Kreutz et al. demonstrated that conversion of 25(OH)D_3_ into 1,25(OH)_2_D_3_ is impaired in GvHD patients and that 25(OH)D_3_ serum levels were lower in grade III-IV than in grade I-II GvHD patients (31). The high prevalence of low vitamin D3 levels in patients undergoing HSCT is reported in other studies as well and might also be associated with a higher incidence of GvHD (32–34). These findings suggest a pivotal protective role of vitamin D_3_ in GvHD pathogenesis. Recently, Chen and Mayne reviewed the immunomodulatory effects of vitamin A and D in the context of GvHD (35). In the following, vitamin D_3_ will be analyzed briefly as an important modulator of both innate and adaptive immune system.

### Molecular Actions of Vitamin D_3_ in the Innate Immune System of GvHD Patients

#### Antimicrobial Activities

Although the precise mechanisms of vitamin D_3_ remained unclear for a long time, patients infected with *Mycobacterium tuberculosis* (*Mtb*) have been treated with UVB irradation and cod liver oil in the pre-antibiotic era (36, 37). In 1980, Rook and colleagues could evidence that growth of *Mtb* was impeded *in vitro* by 1,25(OH)_2_D_3_ in human monocytes and macrophages (38, 39). Since then, it became increasingly clear that vitamin D_3_ exerts anti-microbial effects (40). Subsequent studies demonstrated that 1,25(OH)_2_D_3_ leads to release of anti-microbial peptides such as LL-37 and β-defensin (41–43). LL-37 is the cleavage product of human cathelicidin antimicrobial peptide (hCAP18, CAMP) and is known for its antibacterial function by inducing bacterial lysis and death (44). Upon infection, lung epithelial cells locally produce 1,25(OH)_2_D_3_ which in turn enhances LL-37 expression (45). Cathelicidin-deficient mice have been shown to be more susceptible to infections with *Streptococcus, Pseudomonas*, and *E. coli* (46). Cathelicidin does not only increase phagocytic activity of macrophages (47) but also promotes reactive oxygen species (ROS) production (48, 49), leading to direct antimicrobial effects. Moreover, cathelicidin triggers autophagy and reactivates phagolysosomal fusion in macrophages, which enhances degradation of intracellular pathogens such as *Mtb, Salmonella*, and *Coxiella* (14, 50). Even viral infections with influenza A (51) or fungal infections with *Candida albicans* (52) in mice are reduced by cathelicidin. Accumulating data have revealed that the intestinal barrier is supported by vitamin D_3_-dependent upregulation of tight junction proteins (53, 54), which is a fundamental requirement for efficient defense against pathogens. The loss of intestinal barrier function is also considered to be a driving factor for GvHD development (55). Thus, vitamin D_3_-dependent release of cathelicidin and the protection of epithelial barriers might improve graft- vs.-infection (GVI) effects in allo-HSCT patients.

Recent studies have now discovered a novel role of LL-37 in cancer (56) and inflammatory diseases (57). Strikingly, LL-37 does not only possess anti-microbial but also anti-inflammatory features, since it has been shown to inhibit the release of pro-inflammatory mediators such as TNF-α, IL-6, and IL-8 by neutrophils (48). Additionally, cathelicidin reduces mortality in mice infected with *P. aeruginosa* by neutralizing endotoxin-mediated inflammation (58).

Hence, vitamin D_3_-triggered activity of cathelicidin links anti-microbial and anti-inflammatory effects in the innate immune system. Since GvHD patients have an increased risk for severe infections due to immunosuppressive drugs (59), vitamin D_3_-mediated enhancement of antimicrobial defense mechanisms might reduce co-morbidity by infectious diseases. Therefore, it is conceivable that vitamin D_3_ might play an important and yet unrecognized role in GVI.

#### Anti-inflammatory Effects

As already mentioned, vitamin D_3_ elicits not only antimicrobial but also anti-inflammatory responses. Even though vitamin D_3_ enhances the maturation of human macrophages and their function as phagocytes (60), their capacity of antigen presentation and consequently also the priming of T cells is limited due to reduction of MHC-II expression (30, 61). Instead, 1,25(OH)_2_D_3_ polarizes macrophages toward an anti-inflammatory M2 subtype, which restrains colitis in mice (62). In humans and mice, vitamin D_3_ generates a tolerogenic phenotype and alters the cytokine and chemokine profile of mature DCs (mDCs) *in vivo* and *in vitro*, which are inhibited in differentiation, maturation and proliferation (63–65). In mixed lymphocyte reactions, proliferation of T cells, co-cultured with these 1,25(OH)_2_D_3_-induced tolerogenic DCs, was indirectly inhibited. Apart from preventing DCs to home into the lymph node by reducing CCR7-expression, vitamin D_3_ also decreases expression of the co-stimulatory molecules CD40, CD80 and CD86 and secretion of cytokines such as IL-6, IL-12, and TNF-α (66). Recently, Saul and colleagues revealed that CD31 is increasingly expressed on DCs, leading to impairment of cell-cell contact, which is essential for T cell priming (67). Moreover, secretion of immunosuppressive IL-10 is enhanced while IL-12 secretion by DCs is impaired, which leads to a weaker T helper Th1- and Th17- cell immune response (68). As a result, activation and differentiation of alloreactive CD4^+^ T cells is reduced *in vitro* (65). Furthermore, vitamin D_3_-treated DCs increase the frequency of suppressive CD4^+^CD25^+^${\text{FoxP}}_{3}^{+}$ regulatory T cells (Treg) (69), which fosters peripheral tolerance to allografts (70). One study indicated that vitamin D_3_-mediated increase of CD4^+^${\text{FoxP}}_{3}^{+}$Nrp-1^+^ cells ameliorates collagen-induced arthritis (71). Recently, Xu and colleagues established engineered DCs to *de novo* produce calcitriol in order to generate more gut-homing Tregs for efficient mitigation of intestinal inflammation (72). These results proved that 1,25(OH)_2_D_3_-induced tolerogenic DCs modulate T cells toward a regulatory and anti-inflammatory immune response *in vivo* and ameliorate acute GvHD (aGvHD) in mice (64, 73). Coussens and colleagues suggest that vitamin D_3_ supplementation in tuberculosis patients helps to restrict inflammatory responses by reducing circulating concentrations of chemokines such as CXCL9, CXCL10, and MMP-9 (74, 75). Additionally, upregulation of chemokine receptor CXCR3 fosters DC migration to inflammation spots (69). *In vitro* studies showed that Janus kinase/signal transducers and activators of transcription (JAK/STAT) signaling and inflammatory cytokines, such as IFN-γ, TNF-α, and Flt-3L, are significantly reduced in NK-cells upon vitamin D_3_-treatment or its analog seocalcitol (EB1089) (76). Interestingly, JAK1/2 have already been identified as potential therapeutic targets in GvHD since it was shown to reduce GvHD in mice while GvT could be preserved (77). Clinical trials verified that the JAK1/2 inhibitor Jakafi^®^ (Ruxolitinib) reduces efficiently steroid-refractory GvHD (78, 79) and has recently been approved by the U.S. Food and Drug Administration (FDA).

Altogether, vitamin D_3_ modifies the innate immune system by exerting not only anti-microbial but also anti-inflammatory functions. Since GvHD patients often show co-morbidity of fungal, viral and bacterial infections (80, 81), increased infection rate as well as exaggerated inflammation are key issues needed to be combatted in this disease. Since persistence of APCs despite the conditioning regimen is the major cause of generation of alloreactive lymphocyte, manipulation of the innate immune system toward tolerogenic host-DCs by vitamin D_3_ in order to reduce their allo-stimulatory potential might help to prevent GvHD (82).

### Effects of Vitamin D_3_ on the Adaptive Immune System

Apart from the above discussed indirect effects on T cells by vitamin D_3_-dependent modulation of innate immune cells, the hormone has also direct impacts on the adaptive immune system since T cells are known to express VDR, which enables them to respond to 1,25(OH)_2_D_3_ (83). Although the VDR appeared to be upregulated in activated alloreactive T cells indicating a role of vitamin D_3_ in T cell activation (30), studies proved that 1,25(OH)_2_D_3_ directly inhibits proliferation and IL-2 production of CD4^+^ T cells (84, 85). Similar to its effect on APCs, 1,25(OH)_2_D_3_ reduces expression of homing receptors such as CCR10 as well as secretion of IFN-γ and IL-10 by T cells (86). Especially Th1 cell proliferation is inhibited via the JAK/STAT signaling pathway (87), while Th2 cells are increased directly (88, 89). Therefore, vitamin D_3_ alters the T cell immunity by transforming Th1- and Th17-responses toward an anti-inflammatory Th2-activity. This mechanism is even amplified since expression of CYP27B1 is also enhanced in activated lymphocytes (30). CD8^+^ T cells are inhibited in proliferation *in vitro* and *in vivo* by vitamin D_3_ (90). It is documented that vitamin D_3_ inhibits pro-inflammatory T cells in IBD patients (91). Since IBD pathogenesis is driven by loss of intestinal barrier function, clinical manifestations of IBD resemble GvHD symptoms in the gastrointestinal tract (55). Such parallels suggest that vitamin D_3_ might achieve similar effects in allo-HSCT. However, 1,25(OH)_2_D_3_ does not only affect T cells but also modulates differentiation and antibody-production of B cells (92). In addition, it induces apoptosis and cell cycle arrest of proliferating B cells resulting in impaired plasma cell differentiation and less autoantibody expression (93).

Altogether, vitamin D_3_ has an overall anti-microbial and anti-inflammatory effect on both innate and adaptive immune system. Therefore, vitamin D_3_ could be a potent supplementary agent in GvHD patients which might improve the patient's life quality by decreasing infectious- and inflammation-mediated co-morbidity.

## Potential GvL-effects Mediated by Vitamin D_3_

As mentioned earlier, it is pivotal to preserve the immune balance by avoiding alloreactivity of donor T cells against healthy tissue while still maintaining their anti-tumorigenic effect. Interestingly, vitamin D_3_ does not only reduce harmful GvHD effects but also exerts anti-tumor activity. So far, scientific literature supporting this assumption in the transplantation setting remains sparse. However, a few studies provide indications for its hypothetical anti-cancer effects. In their retrospective study, Radujkovic et al. could demonstrate that pre-transplant vitamin D_3_ deficiency in patients diagnosed with myeloid malignancies correlates with a higher risk of relapse mortality (94). To our knowledge, only three other studies have also investigated this association (90, 95, 96). So far, only one study included few patients which underwent autologous transplantation (97). However, they only figured out that sufficient vitamin D_3_ levels are hard to achieve. In summary, these data suggest that prospective randomized trials have to prove whether vitamin D_3_ supplementation during stem cell transplantation could enhance GvL effects.

### Vitamin D_3_ and Cancer

The first correlation of solar radiation and cancer was initially suggested by Apperly in 1941, who attributed sunlight radiation a protective role against many types of cancer except skin cancer (98). Decades later, Colston et al. were the first ones to show a dose-dependent inhibitory effect of 1,25(OH)_2_D_3_ on melanoma cells (99, 100). Epidemiological studies provide evidence that poor sunlight exposure and vitamin D_3_ deficiency correlate directly with incidence as well as mortality rate of several cancer types. These findings suggest a protective role of vitamin D_3_ in carcinogenesis (101). Accumulating studies have revealed that 1,25(OH)_2_D_3_ suppresses tumor growth (102–104) and exhibits anti-proliferative activities in squamous cell carcinoma (105), prostate (106), breast (107, 108), lung (109), head and neck cancer (110) and hematologic malignancies such as Hodgkin's lymphoma (111) or chronic lymphocytic leukemia (CLL) (112). In colorectal cancer, a clinical trial provides evidence that 1,25(OH)_2_D_3_ supplementation can efficiently reduce the risk of tumor development (113). However, other epidemiological studies report contradictory results (114–116), which might be a result of using supra-physiological concentrations of calcitriol (117), VDR gene polymorphisms (118), lack of control groups or inappropriate dosage and administration of vitamin D_3_.

Apart from the discovery that sufficient vitamin D_3_ supplementation could help to prevent cancer pathogenesis, numerous *in vitro* and *in vivo* studies provide evidence that 1,25(OH)_2_D_3_ and its analogs could reduce tumor growth and might be used as potential anticancer agent (15, 119–121). Supporting this, animal studies report that VDR-deletion in mice makes them more susceptible to chemical induced carcinogenesis in epidermis, lymphoid and mammary tissue (122). Interestingly, life expectancy of leukemic mice could be prolonged by treatment with a 1,25(OH)_2_D_3_ analog (123). A chemoprevention study revealed that 1,25(OH)_2_D_3_-treated *Nkx3.1;Pten* mutant mice show retarded development of neoplasias when it was administered during early-stage carcinogenesis (124).

There is clear evidence that cancer cells exploit and dysregulate the vitamin D_3_ metabolism enabling them to escape its cancer protective role (15). CYP24A1 has been shown to be overexpressed in cancer cells while activity of CYP27B1 is reduced in human prostate cancer cells (125, 126). Furthermore, CYP24A1 was identified as potential oncogene in breast cancer and elevated expression of VDR in tissues of breast and prostate cancer correlates with better prognosis of survival (127).

### Mechanisms of Anti-tumorigenic Actions

Although the precise mechanisms of vitamin D_3_-mediated anti-tumorigenic action are not yet fully understood, it has been postulated that vitamin D_3_ modulates gene expression involved in apoptosis, cell cycle and autophagy in tumor cells (128). Apoptosis is initiated due to downregulation of anti-apoptotic protein Bcl2 while expression of pro-apoptotic proteins increases (129). Jiang et al. suggest that 1,25(OH)_2_D_3_ induces cell death by degrading telomerase reverse transcriptase (TERT) mRNA and thus reduces telomerase activity (130). 1,25(OH)_2_D_3_-induced upregulation of p21 and p27, which are cyclin-dependent kinase (CDK) inhibitors, induces cell cycle arrest (121, 129, 131). Furthermore, vitamin D_3_ mediates anti-proliferative activity by enhancing expression of Dickkopf-1 (DKK-1), which is an antagonist in the Wnt/β-catenin signaling pathway (132). *In vitro* as well as *in vivo* studies report inhibition of proliferation and angiogenesis by vitamin D_3_. It suppresses hypoxia-inducible factor 1-alpha (HIF1A) leading to reduced expression of vascular endothelial growth factor (VEGF) and thereby inhibition of angiogenesis (133, 134). Autophagy is not only triggered in infected macrophages, but also in tumor cells such as breast cancer. Since autophagy appears to protect healthy tissue from cancer initiation, vitamin D_3_-treatment might contribute to suppression of carcinogenesis (135). It also increases activity of antioxidant enzymes such as superoxide dismutase 1/2 (SOD1/2) and therefore protects DNA from ROS-induced damage (129). Upon vitamin D_3_ administration, DNA damage repair proteins, such as p53, are upregulated *in vitro* (15).

Strikingly, anti-tumor activity of tumor-associated macrophages (TAMs) against lymphomas has been shown to be enhanced by vitamin D_3_-triggered increase of antibody-dependent cellular toxicity (ADCC) and antibody-dependent cellular phagocytosis (ADCP) (136). Current observations of Busch et al. reveal that combination of vitamin D_3_ with immunomodulatory drugs (IMiDs), such as lenalidomide, helps to restore the defective vitamin D_3_ metabolism in myeloma-associated macrophages and improves cytotoxicity against multiple myeloma cells mediated by specific anti-CD38 antibodies such as MOR202 (137, 138). Furthermore, exosomal transfer of microRNAs, which induce tumor-promoting myeloid-derived suppressor cells, was impeded by vitamin D_3_ (139). The previously mentioned cathelicidin, which is secreted by human macrophages, has also been shown to mediate direct anti-tumor efficacy against high-grade B cell lymphoma by increasing ADCC (136). In summary, there is strong evidence that vitamin D_3_ exerts direct anti-tumorigenic functions which might be applicable in allo-HSCT patients in order to boost GvL effects.

### Mediation of Anti-inflammation to Antagonize Carcinogenesis

In 1863, Virchow postulated for the first time that tissue proliferation and hence tumor progression might be provoked by an inflammatory microenvironment connecting cancer with inflammation (140, 141). Since inflammatory tissue provides ideal conditions for genetic mutations, it seems obvious that tumor progression occurs more frequently in inflammatory environment than in healthy tissue. Clinical studies proved that localized persistent inflammation is a risk factor for the development of cancer in adjacent organs, e.g., patients with ulcerative colitis have a higher incidence of colorectal cancer (142). Given that inflammation promotes carcinogenesis, vitamin D_3_-dependent anti-inflammatory activity could reduce tumor progression. In esophageal squamous cell carcinoma, 1,25(OH)_2_D_3_ impedes tumor growth by inhibition of IL-6 signaling (117). Accumulating data report that 1,25(OH)_2_D_3_ inhibits prostaglandin (PG) (143), p38 MAPK (144) and nuclear factor kappa B (NFκB) signaling pathways (15). Although there is increasing evidence for inflammation-driven carcinogenesis, not every type of chronic inflammation evokes tumor development, which appears to be contradictory.

Despite the well-founded evidence of anti-tumorigenic effects of vitamin D_3_ in solid tumors, studies on hematological malignancies remain elusive. Given that vitamin D_3_ deficiency correlates with worse relapse-free survival (94–96) and the known anti-tumorigenic effects of vitamin D_3_, one might think that it could also enhance GvL. By using mice fed with low and high vitamin D_3_ doses or by performing clinical trials with vitamin D_3_ supplementation, the actual effect on GvL could be investigated.

## Conclusion/Perspectives

**Figure 2:**
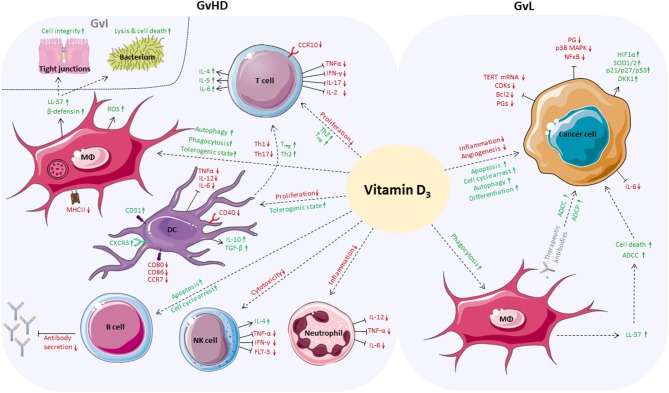
Positive effects of vitamin D_3_ in the allo-HSCT setting. Vitamin D_3_ affects a broad variety of cells of the adaptive and innate immune system which play an important role in boosting GvL responses while alleviating GvHD. On side of GvHD, proliferation of T cells is inhibited and anti-inflammatory differentiation is favored. Vitamin D_3_ enhances autophagy, phagocytic activity and tolerogenic capacity of macrophages. Vitamin D_3_-dependent release of antimicrobial peptides by macrophages fortifies GvI effects, increases cell integrity, and induces cell death of bacteria. DCs are inhibited in proliferation and reduce Th1 and Th17 responses while the T_reg_ population increases. Vitamin D_3_ triggers apoptosis and cell cycle arrest of B cells and inhibits antibody secretion. Vitamin D_3_ reduces cytotoxic activity of NK cells and impedes neutrophils to secrete pro-inflammatory cytokines. In case of GvL, apoptosis, autophagy and differentiation of cancer cells are directly enhanced by vitamin D_3_, whereas inflammation and angiogenesis are reduced. Vitamin D_3_ enhances ADCC and ADCP of cancer cells by macrophages directly or by release of LL-37.

In summary, we assume that vitamin D_3_ could be a potential immune modulating agent for supplementation before and during allo-HSCT. It is conceivable that vitamin D_3_ might be able to maintain and improve the patient's immune balance and epithelial barrier function. Mounting evidence indicates that vitamin D_3_ could alleviate GvHD by enhancing anti-inflammatory responses while it might coincidently ameliorate GvI effects due to its anti-microbial activities. Moreover, GvL might be boosted because vitamin D_3_ could at least reinforce anti-tumorigenic responses of myeloid cells (Figure 2).

Besides its easy availability, economy and role in preserving the intestinal barrier integrity (53), vitamin D_3_ helps to maintain calcium and bone homeostasis and hence prevents osteoporosis. Given that allo-HSCT patients often suffer from bone loss upon conditioning regimens, immunosuppressive treatment and immobilization, it might also improve GvHD by preventing osteoporosis (34). Cholecalciferol can usually be administered safely in high doses without occurrence of abnormal calcium metabolism (145). However, sufficient vitamin D_3_ levels cannot be achieved in all patients despite high-dose supplementation (97). Therefore, treatment with 1,25(OH)_2_D_3_ might be the more efficient version. However, the probably greatest restraining factor of 1,25(OH)_2_D_3_ is its dose-limiting toxicity causing hypercalcemia and hypercalciuria. One possible solution might be the administration of 1,25(OH)_2_D_3_ analogs, some of which have already been shown to be less calcemic (146, 147).

Until now, only few clinical trials with vitamin D_3_ in the allo-HSCT setting have been conducted and have shown effective outcomes (90). The most recent study of Carillo-Cruz et al. suggests that universal vitamin D_3_ medication remains challenging due to VDR polymorphisms (29). Our hypothesis that vitamin D_3_ could improve GvL might seem controversial due to its known anti-inflammatory activities on T cells. However, it was shown by Essen et al. that TCR signaling in naïve human T cells induces VDR expression (148). This in turn results in upregulated PLC-γ1 expression and thus higher activation and priming of naïve T cells. Although there is evidence that vitamin D_3_ attenuates IL-6 signaling in human esophageal squamous cell carcinoma (SCC) cell lines (117), the *in vivo* study of Bendix-Struve and colleagues demonstrated that T cells of vitamin D_3_-supplemented Crohn's disease (CD) patients produced more IL-6 (149). Proliferation of CD4+ T cells was higher in vitamin D_3_-treated patients compared to the placebo group. Additionally, VDR was shown to be important for the development of CD8αα+ TCRαβ+ cells, which help to maintain tolerance in the gut and suppress intestinal inflammation (150). Expression of the gut-homing receptor CCR9 is suppressed in T cells upon vitamin D_3_ stimulation, which might prevent homing of potential alloreactive T cells into the gut. However, these data provide only indications that vitamin D_3_ might promote GvL despite its anti-inflammatory properties.

In conclusion, prospective *in vivo* studies in humans are inevitable to investigate the efficacy of vitamin D_3_ supplementation and to achieve approved clinical application.

## Author Contributions

CF wrote the manuscript and created the figures. HB took the lead in writing the manuscript. MK and KP provided critical feedback, contributed to the final version of the manuscript, and approved it for publication.

### Conflict of Interest

The authors declare that the research was conducted in the absence of any commercial or financial relationships that could be construed as a potential conflict of interest.
